# *Chlamydia trachomatis* Infection as a Risk Factor for Ovarian Carcinoma †

**DOI:** 10.3390/microorganisms14081637

**Published:** 2026-07-27

**Authors:** Mirja Puolakkainen, Jorma Paavonen, Robert C. Brunham

**Affiliations:** 1 Department of Virology, University of Helsinki, 00014 Helsinki, Finland; 2 University of Helsinki, 00014 Helsinki, Finland; 3 Department of Medicine, British Columbia Centre for Disease Control, University of British Columbia, Vancouver, BC V5Z 4R4, Canada

**Keywords:** *Chlamydia trachomatis*, epithelial ovarian carcinoma, serology

## Abstract

*Chlamydia trachomatis* (CT) is the most common global cause of bacterial sexually transmitted infection. CT is a common cause of mucopurulent cervicitis and pelvic inflammatory disease. This review examines the epidemiologic, serologic, mechanistic, and meta-analytic evidence linking CT infection as a risk factor for ovarian cancer. Further study of the role of CT infection in ovarian cancer risk is warranted.

## 1. Background

Infection-associated chronic inflammation likely contributes to many human epithelial cell cancers [1,2] by causing genomic instability and multiple types of DNA modification [3,4]. *Chlamydia trachomatis* (CT) infects epithelial cells and is the most common global cause of bacterial sexually transmitted infection [5]. The most distinctive microbiological characteristics of CT are its biphasic developmental cycle, best seen in tissue culture, and its tendency to produce persisting infection, best seen in infected humans [6]. CT is serologically variant with at least 15 different serovars, which can be clinically correlated with different disease phenotypes. Serological variants or serovars A through C cause the ocular disease trachoma; serovars D through K cause a variety of reproductive tract infection syndromes; and serovars L1 through L3 cause the systemic infection syndrome lymphogranuloma venereum. CT’s unique biphasic developmental cycle involves infectious but non-replicative elementary bodies (EBs), which transmit between hosts and enter host cells, where they transform into non-infectious replicative reticulate bodies (RBs). After replication, RBs convert back to EBs, which are released from the host cells to initiate new rounds of infection. Importantly to disease pathogenesis, RBs can transform into non-replicative, aberrant bodies, which allow the bacteria to persist inside cells for extended periods of time, where they can cause inflammation and modify host cell biology [7,8].

The cervix is the initial site of CT infection and comprises an ectocervix lined by a stratified squamous epithelium that projects into the vagina and an endocervix lined by a columnar epithelium. The ecto- and endocervix merge at the squamocolumnar transformation zone. Mucosal surfaces of the female reproductive tract play an essential role in protecting against infection. Since it is critical to maintain conditions suitable for fertilization and implantation in the upper female reproductive tract, the lower female reproductive tract, particularly the cervix, acts as the major anti-microbial barrier between the lower and upper genital tract. The vagina, with its lactobacillary microbiome, maintains acidity and limits pathogenic microbial colonization [9,10]. CT can overcome these barriers and spread from the lower to the upper genital tract [11].

The host immune response to CT is complex, extending from a complete clearance of infection to prolonged persistence and severe inflammation [6,12]. Clinically, CT is the major cause of mucopurulent cervicitis and pelvic inflammatory disease (PID) [13,14]. Long-term sequelae of chlamydial PID include hydrosalpinx formation, tubal factor infertility and ectopic pregnancy [14]. CT serology is commonly used to define prior infection, and nucleic acid amplification tests are used to define active infection. Such studies have linked CT to epithelial ovarian cancer (EOC) [15].

Ovarian cancer is the most lethal of all gynecological cancers. Multiple histologic types of ovarian cancers are recognized by pathologists, with low-grade and high-grade serous ovarian cancer (HGSOC) comprising the majority of epithelial ovarian cancer (EOC) types [16]. Established risk factors for EOC include nulliparity, early menarche, late menopause, endometriosis, germ line BRCA1 or BRCA2 mutations, Lynch syndrome, and smoking. Conversely, oral contraceptive use, parity, tubal ligation, salpingectomy, and hysterectomy reduce the risk of EOC [17]. Emerging and compelling evidence suggests that HGSOC arises from epithelial secretory cell precursor lesions in the fallopian tubes [18]. The loss of tubal ciliated epithelium is a precancerous feature, with the number of ciliated cells decreasing with age and in women at high risk of HGSOC [19]. Interestingly, CT infection of the fallopian tube also causes a loss of ciliated epithelial cells [14,20,21], and chronic salpingitis has been detected in up to half of EOC cases [22]. Normal-appearing tubal secretory cells with mutations in tumor suppressor gene p53 and serous tubal intraepithelial carcinoma (STIC) are considered as precursors of HGSOC [19]. Importantly, STIC cells in the fallopian tube can detach and seed the ovarian surface [18]. Such cells may subsequently undergo further neoplastic transformation.

## 2. Epidemiological Evidence Linking *Chlamydia trachomatis* to Ovarian Cancer

CT infection has been epidemiologically implicated as a potential risk factor for EOC in many [23,24,25,26,27] but not all studies [28,29,30]. Both antibody detection and nucleic acid detection assays have been used in these studies. A recent systematic review and meta-analysis [15] including 11 observational studies involving 4518 participants revealed an overall non-significant association between CT infection and ovarian cancer risk. However, after excluding two studies, both sensitivity analyses and subgroup analyses based on control group type (healthy controls) demonstrated a significant overall association. The studies included exhibited significant heterogeneity in their methodologies. Variations included the use of mixed control groups (comprising both benign conditions and healthy women), differences in diagnostic techniques (serology versus molecular methods), and adjustments for numerous covariates (such as parity, smoking, and BMI). This variability may help explain the inconsistent findings seen across the original studies.

Heterogeneity in serological methods used could be a major contributing factor for the varying epidemiological findings. In studies relying on microimmunofluorescence or ELISA serology with purified whole bacteria as antigens, no association with primary fallopian tube carcinoma or ovarian tumor of any type was noted [28,29,31]. However, when antibodies against chlamydial plasmid-encoded protein 3 (Pgp3) or heat shock protein 60 (Hsp60) were detected, an elevated risk of EOC was observed in several studies [23,24,25,26,31] but not in all [29,30]. Measuring antibodies against specific CT proteins instead of whole bacteria seems more reliable and reproducible as a marker of prior exposure to CT and tissue damage and thus more suitable for these analyses. In addition, a recent Mendelian randomization study identified an association between genetically predicted CT major outer membrane D (momp D) seropositivity and HGSOC [32]. Thus, both the serological assay used in detecting CT antibodies and the histologic type of ovarian cancer studied may contribute to the heterogeneity in the serological associations found.

Lastly, CT DNA has been detected in cancerous ovarian tissue in three small studies [33,34,35]. However, a larger study using more standard methods for detecting CT DNA did not detect CT nucleic acid in any of the ovarian tissue samples examined [36]. Clearly, additional studies with appropriate sample size and histologically defined ovarian cancer types that use standard CT DNA detection assays will be needed to determine whether persistent CT infections are associated with ovarian cancer risk. Such studies should also include standardized assays for detecting CT Pgp3 and Hsp60 antibodies.

## 3. Basic and Translational Evidence Linking *Chlamydia trachomatis* Infection to Ovarian Cancer

Several lines of basic and translational research evidence suggest that CT infection may play a role in ovarian cancer pathogenesis. Infection promotes epithelial-to-mesenchymal transition, a hallmark of cancer progression [37]. During infection, CT proteins inhibit apoptosis by blocking cytoplasmic caspase activation and mitochondrial cytochrome C release [38], allowing damaged cells to survive and proliferate. CT induces centrosome amplification, another hallmark of cancer cells [39]. Additionally, CT infection promotes genomic instability by inducing inflammatory cells to release reactive oxygen species (ROS) and impede DNA repair mechanisms [40,41], including diminished activity of key tumor suppressor genes, such as p53 [42,43]. CT can also induce the expression of matrix metalloproteinases, which degrade extracellular matrix components and facilitate epithelial cell migration and tumor invasion [44].

**Figure 1:**
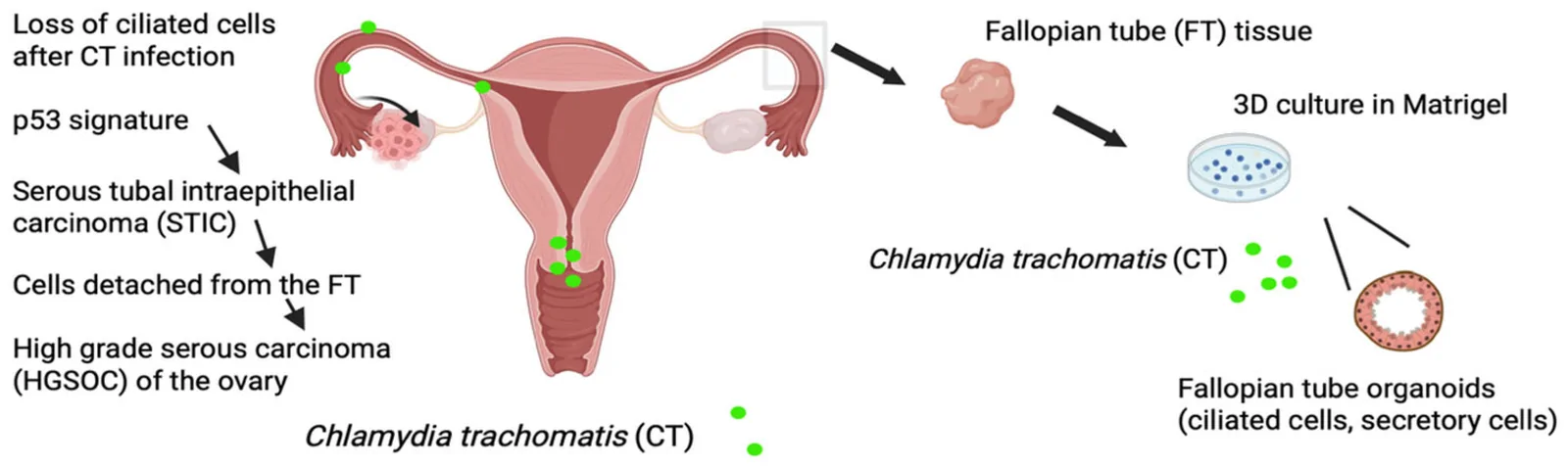
Potential effects of ascending *Chlamydia trachomatis* infection associated with carcinogenesis (left side) [19,47,49,51]. Right side shows a model based on fallopian tube organoid cultures. Infected organoids can model chronic CT infection and its long-term effects, including potential to contribute to cell transformation [45,47]. Created in Biorender. Puolakkainen, M. (2026) https://app.biorender.com/illustrations/canvas-beta/691b185f80bf288fa71c28b6.

The recent development of the three-dimensional organoid model system from the female genital tract enabled detailed cellular and molecular studies of cervical and fallopian tube CT infection [45,46] (Figure 1). These advanced organoids closely replicate native tissue architecture, maintain genetic and phenotypic stability, and support long-term CT infection. The fallopian tube organoid infection model demonstrates how CT may act as a co-factor in the development of HGSOC.

Using the model, Kessler et al. identified sustained CT-driven changes in cellular differentiation of the epithelium over the course of fallopian tube infection [47]. Approximately 30% of cells were infected. Notably, the extrusion of EB-rich vacuoles into the lumen of the organoid enabled the infection cycle to continue for up to 3 months without destruction of the epithelial layer. Gene expression profiles in the organoids revealed robust IFN-β signaling as the most prominent hallmark of acute infection [47]. The absence of caspase-3 activation confirmed that infected cells did not die by apoptosis [47]. Persistent CT infection in fallopian tube organoids led to a gradual decrease in the number of ciliated cells, a hallmark of CT-induced salpingitis in human infection [14,21]. This may represent a link to cell transformation, since, as previously mentioned, the expansion of non-ciliated secretory cells precedes HGSOC development. Persistent CT infection in fallopian tube organoids was accompanied by increased stemness of the epithelial layer associated with increased CpG methylation of the host cell DNA [47]. Consistently upregulated genes in CT-infected fallopian tube organoids were leukemia inhibitory factor (LIF) and osteopontin [47]. LIF is a pleiotropic cytokine induced by inflammatory stimuli that plays a role in promoting cancer stem cells and epithelial-to-mesenchymal transition [48,49]. Osteopontin is upregulated in HGSOC [50] and supports epithelial cell migration and epithelial-to-mesenchymal transformation [51]. Osteopontin also remodels extracellular interactions, further biasing toward a tumor-promoting micro-environment [51]. Thus, persistent CT infection establishes conditions that may be conducive to HGSOC initiation by leveraging LIF and osteopontin signaling to reprogram the fallopian tube epithelium.

## 4. Conclusions

Epidemiological and mechanistic evidence of an association between CT infection and ovarian cancer suggests a causal relationship. Some cohort and case–control studies report modest associations (RR/OR ~1.3–2.3) between CT infection and ovarian carcinoma, suggesting that there is an association, but it may be weak. Meta-analyses, however, show significant study heterogeneity. Variability stems from differences in CT biomarkers used, histologic types of ovarian cancer studied, study inclusion criteria, type of control group, timing of infection, and residual confounding. Advances in CT sero-epidemiology, with the use of Pgp3 and Hsp60 antigens to measure IgG1 and IgG3 antibody responses, have significantly improved the reliability and reproducibility of CT serology [52,53,54,55]. These assays are now available for prospective serological studies to evaluate the association of CT infection with ovarian cancer.

New data on the molecular mechanisms by which CT triggers oncogenesis are essential for supporting the association between CT infection and cancer. The development of epithelial cell organoids from the female reproductive tract [45] and multi-omics of the cancerous tissue [56] are major experimental systems that are now available for exploring this association. Further large-scale studies using these methods to study the relationship between CT infection and ovarian cancer are recommended. Finally, evaluating the impact of CT control programs on ovarian cancer incidence is important, though any effect may take decades to become evident. Among US registry data, HGSOC incidence has significantly declined between the 1990s and 2019 across ethnic/racial groups [57]. This decline in incidence has been attributed to the wider use of oral contraceptives, but this is also the era in which CT control programs have expanded. Comparative analyses between regions with long-standing versus newly implemented CT control programs could offer insight into long-term cancer prevention benefits.

## Data Availability

No new data were created or analyzed in this study. Data sharing is not applicable to this article.

## Abbreviations

The following abbreviations are used in this manuscript:

CT	Chlamydia trachomatis
EB	Elementary body
EOC	Epithelial ovarian cancer
HGSOC	High-grade serous ovarian cancer
Hsp60	Heat shock protein 60
LIF	Leukemia inhibitory factor
Pgp3	Chlamydial plasmid-encoded protein 3
PID	Pelvic inflammatory disease
RB	Reticulate body
ROS	Reactive oxygen species
STIC	Serous tubal intraepithelial carcinoma
